# The vesicle-associated function of NOD2 as a link between Crohn’s disease and mycobacterial infection

**DOI:** 10.1186/s13099-015-0049-1

**Published:** 2015-01-29

**Authors:** Alexey A Nabatov

**Affiliations:** Maastricht Radiation Oncology, MAASTRO/GROW Maastricht University Medical Center+, PO Box 616, 6200 MD Maastricht, The Netherlands; Science Center, Volga Region State Academy of Physical Culture, Sport and Tourism, 33, Universiade Village, Kazan, 420138 Russia

**Keywords:** Vesicle acidity, Crohn’s disease, Mycobacteria, Sulfonated glycolipids, Autophagy, Pattern recognition receptors

## Abstract

Although Crohn’s disease (CD) etiology remains unclear, a growing body of evidence suggests that CD may include an infectious component, with *Mycobacterium avium* subsp. *paratuberculosis* (MAP) being the most likely candidate for this role. However, the molecular mechanism of the MAP involvement in CD pathogenesis remains unclear. The polymorphism of the *NOD2* gene, coding for an intracellular pattern recognition receptor, is a factor of predisposition to mycobacterial infections and CD. Recent findings on NOD2 interactions and functions provide the missing pieces in the puzzle of a NOD2-mediated mechanism common for mycobacterial infections and CD. Implications of these new findings for the development of a better understanding and treatments of CD and mycobacterial infections are discussed.

## Introduction

Crohn’s disease (CD) is a systemic inflammatory disease involving primarily the intestinal tract and associated with the variety of extraintestinal manifestations. Although it may affect any part of the digestive tract from the mouth to the anus, it most commonly affects the last part of the small intestine (ileum) and/or the large intestine (colon and rectum). The etiology of CD remains unclear. However, there is phenotypical, epidemiological and clinical evidence of *Mycobacterium avium* subsp. *paratuberculosis* (MAP) involvement in CD development [1-3]. This enteric pathogen is significantly associated with CD [4-7]. MAP causes paratuberculosis (Johne’s disease), a chronic, contagious bacterial disease that primarily affects the small intestines of ruminants. Johne’s disease affects approximately 68% and 32% of cows in the USA and the UK, respectively [7,8]. Live MAP is found even in pasteurized cow’s milk, suggesting that dairy products and beef, widely present in the “western” diet, can play a role in MAP transmission to human population [1]. The lack of evidence for horizontal or vertical transmission of CD suggests that MAP is a zoonotic agent or an opportunistic pathogen in humans [9]. The signs of Johne’s disease in ruminants are similar to the symptoms of CD. Moreover, CD demonstrates a striking similarity of symptoms to intestinal tuberculosis caused by *M. tuberculosis*, Mtb [10-12].

The systemic characters of CD and mycobacterial infections suggest that the underlying pathological processes are defects in basic cellular signaling mechanisms common to different cell types. However, these molecular mechanisms remain unclear. As a result, CD remains incurable and its incidence increases around the globe, which makes CD a global health problem with high societal costs and a substantial health-related quality-of-life burden [13,14]. The recent rapid growth of CD incidence in Asia may be related to the westernization of the diet and an improved hygiene [15].

The polymorphism of Nucleotide binding and Oligomerization Domain 2 (*NOD2)* is a genetic predisposition factor for both CD and mycobacterial infections [16-20]. However, it does not seem to contribute significantly to the CD incidence in Eastern Asians, probably due to the low presence of the characteristic CD-associated NOD2 polymorphisms in this part of the world (Rs2066844; Rs2066845; Rs2066847 (Rs5743293)) [15,21,22]. These facts suggest that NOD2 polymorphism is rather secondary for disease development, which, however, does not exclude a NOD2 role in CD etiology.

It is prompting to speculate that NOD2 mediates a mechanism important for both mycobacterial infection and CD. However, until recently, little was known about what basic NOD2-dependent mechanism could link CD and mycobacterial infection and at the same time explain the characteristic features of these diseases. Several years ago it became clear that, to prove the mycobacterial hypothesis of CD, immunologists should identify the microbe-associated ligands mediating CD immune defects [23]. In the last two years, this gap in understanding of CD etiology has been filled for NOD2. This review for the first time summarizes the new findings linking NOD2, mycobacterial infection and CD development, and explains some characteristic molecular features of these diseases.

### NOD2 and its ligands

The *NOD2* (*Blau, CARD15*) gene encodes a 115-kDa cytosolic protein with multiple C-terminal leucine-rich repeats (LRR), a central NACHT (NAIP, CIITA, HET-E, TP-1) domain, and two N-terminal caspase recruitment domains (CARDs). The NACHT domain bears high homology to NTPase domains; however, the intrinsic NTPase activity of the NACHT domain is not well established. The NOD2 NACHT domain resembles the ATPase domain of the proton-pumping F1-ATPase, which in turn is highly similar to that of Vacuolar-type H^+^-ATPase (V-ATPase) [24,25]. The NACHT domain mediates homo- and heterotypic oligomerization, which triggers recruitment of pro-inflammatory factors (caspase-1 and RIP2) to CARDs and enhances pro-inflammatory activity at both the transcriptional and the post-transcriptional levels [26-30]. Unbound to a ligand, the LRR domain covers the NACHT domain and prevents the NACHT-mediated oligomerization [29]. The genetic polymorphism of the NOD2 LRR predisposes to CD whereas the NACHT polymorphism is associated with deregulation of NF-kB activity and development of Blau syndrome, an inflammatory disorder that primarily affects the skin, joints, and eyes [16,17,31].

Increased NOD2 expression alone can activate pro-inflammatory NF-kB activity, suggesting a default character of this NOD2 activity [28,32]. The baseline gene expression of *NOD2* is very low in different cell types, reflecting the specific and powerful characters of the NOD2 regulated processes [33]. Indeed, *NOD2* gene expression is up-regulated under stress conditions such as hypoxia or the presence of bacterial lipopolysaccharides, both known to regulate the transcriptional activity of hypoxia-inducible factor type 1 (HIF-1) [32,34-36].

NOD2 also mediates autophagy, a catabolic intracellular process of partial cytoplasm sequestration into double-membrane autophagosomes that fuse with lysosomes to digest the sequestered material [37,38]. Muramyl dipeptide (N-acetylmuramyl-L-alanyl-D-isoglutamine), a fragment of the bacterial cell wall, seems to be an unspecific NOD2 activator inducing both pro-inflammatory and autophagy activities [26-29,37,38]. The processes of inflammation and autophagy are antagonistic to each other [39]. For NOD2, it may mean that NOD2 mediates inflammation by default if it is not involved in autophagy.

NOD2 belongs to the family of pattern recognition receptors (PRRs) serving as innate immunity sensors. PRRs recognize a limited number of conservative immunogenic epitopes (patterns) including endogenous damage-associated molecular patterns, DAMPs [40-42]. Autophagy-inducing cytoplasmic PRRs can specifically recognize host glycans from the outer leaflets of membranes when membrane damage (i.e. caused by pathogens) exposes the outer glycans to the cytoplasm [43]. These findings may shed additional light on the sentinel role of NOD2 at the host membranes [44].

3-O-sulfogalactocerebroside (sulfatide), a sphingolipid normally present on the outer membrane leaflet, has recently been identified as the first NOD2 DAMP mediating NOD2 involvement in autophagy [32]. Of interest, hypoxia also stimulates the gene expression of *GAL3ST1* (Galactose-3-O-sulfotransferase 1)*,* whose protein product catalyzes the conversion of 3’-phosphoadenosine-5’-phosphosulfate (PAPS) + galactosylceramide to adenosine 3’,5’-bisphosphate + sulfatide [32]. These findings are in line with others showing that renal carcinoma cells, known for their deregulated activity of HIF-1, have elevated sulfatide and sulfotransferase activities [45,46]. Thus, the co-expression of NOD2 and GAL3ST1 prepares vulnerable membranes to effective recognition by NOD2 and subsequent autophagy if the membranes become damaged.

### NOD2 vesicle-associated function

**Figure 1:**
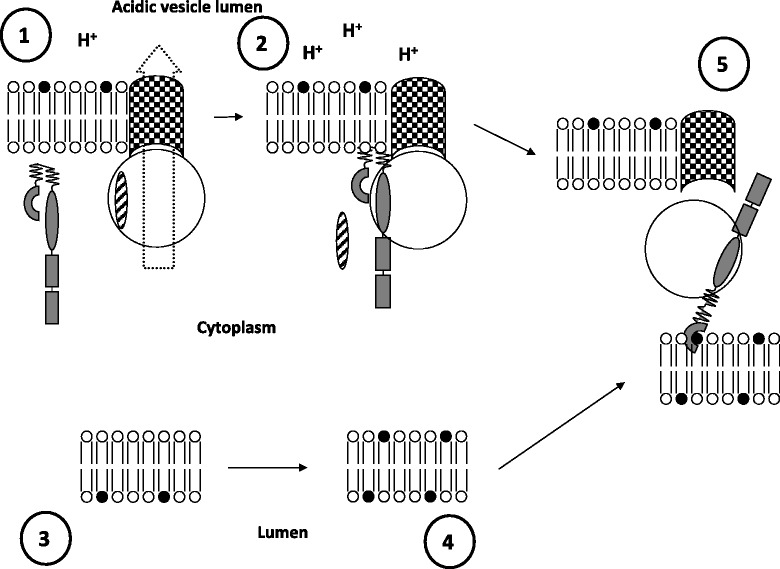
**The model of NOD2 vesicle-associated function.** 1) Catalytically active V-ATPase consisting of transmembrane V0 and cytoplasmic V1 (big circle) sectors pumps (big dotted arrow) protons from the cytoplasm into the vesicle. Cytoplasmic NOD2 (grey figure) is in the self-inhibiting state; 2) NOD2 may substitute for the catalytic V1A subunit (stripped oval) in the V-ATPase complex, when pumping is stopped and the rest of V-ATPase complex remains assembled. 3) A normal membrane keeps sulfatide (black-head “lipid”) on the outer (opposite to the cytoplasm) leaflet. 4) When the membrane is damaged, it exposes sulfatide to the cytoplasm. 5) The sulfatide exposure to the cytoplasm is recognized by NOD2, which induces V-ATPase complex disassembly and opens the fusion-mediating V0 sector, making the vesicle fusion-competent.

Intracellular vesicle-associated acidity grows under hypoxia [47]. This vesicle acidity is mediated by the V-ATPase proton-pumping catalytic activity. These newly formed vesicles need to protect their acidity because the hypoxia-associated ATP deficiency can induce vesicle leakage [48]. However, the V-ATPase function is not limited to proton pumping. Assembled but inactive V-ATPase mediates vesicle content storage, whereas its disassembly mediates vesicle fusion and content release (including leakage) [49-52]. NOD2 deficit decreases the intracellular vesicle acidity but not vesicle acidification, suggesting a NOD2 role in vesicle content storage. NOD2 interacts with assembled, catalytically inactive V-ATPase until the NOD2 – V-ATPase complex reaches sulfatide-rich membranes, where the V-ATPase disassembles (Figure 1) [32]. These and more recent findings directly link NOD2 function to intracellular vesicles [53].

At high ATP concentrations, the catalytic activity of V-ATPase may compensate for the lack of NOD2 functionality by pumping the leaked protons back into the vesicles. This makes NOD2 protein dispensable for normal conditions, which is supported by the very low *NOD2* gene expression in normal conditions. However, stress conditions associated with a deficit of ATP production (e.g. hypoxia) will increase the need for the NOD2-mediated energy-saving mechanism of proton storage in vesicles.

**Figure 2:**
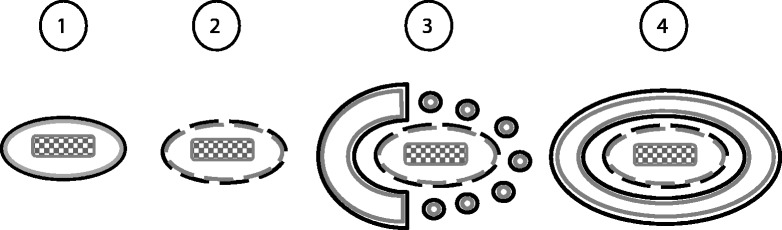
**The activated by NOD2 vesicles in autophagy.** 1) A phagosome containing a microbe (checkered figure) becomes damaged and 2) exposes sulfatide to the cytoplasm. 3) Autophagy is initiated and fusion-competent vesicles are accumulated around the damaged phagosome due to V-ATPase disassembly triggered by sulfatide-NOD2 interaction. 4) The fusion competent vesicles fuse with the autophagosome providing material and directing autophagosome growth specifically around the damaged phagosome. The outer (sulfatide-containing) membrane leaflet is grey; the inner membrane leaflet is black.

The induction of “fusion-competent” vesicles after NOD2-sulfatide interaction suggests their accumulation in close proximity to damaged membranes. These vesicles may provide membrane material and eventually direct autophagosome growth specifically around the damaged membranes, without sequestration of undamaged areas (Figure 2). On the other hand, the presence of sulfatide-mimicking agents at a distance from the sulfatide-exposing membranes will inhibit the specificity of NOD2 function and induce unspecific fusion and vesicle content release (leakage).

### NOD2 and mycobacterial immune escape

Macrophages, professional antigen-presenting immune cells expressing one of the highest amounts of NOD2 in the body, are the preferred hosts for MAP and Mtb*.* Whether active mycobacteria remain inside phagosomes or translocate from phagosomes into the cytosole has been a matter of debate in recent years [54]. In both cases, mycobacteria should perforate the phagosomal membrane to egress into the cytoplasm or to get access to the cytoplasm nutrients [55,56]. Obviously, to survive, mycobacteria induce mechanisms that prevent or subvert the membrane-damage-associated activation of NOD2.

Mtb lipid virulence factors may have evolved to mimic host lipids and thereby directly influence innate immune responses of macrophages via interactions with specific signaling pathways [56]. Mycobacteria synthesize a specific sulfolipid (SL-1) that mimics sulfatide in binding to NOD2 [32]. This suggests that SL-1 interaction with NOD2 can activate unspecific NOD2-mediated processes of V-ATPse disassembly, making intracellular vesicles leaky and/or activating unspecific fusion of these vesicles. This clarifies the SL-1-mediated inhibition of: 1) lysosome fusion with Mtb-containing phagosomes and 2) lysosome maturation [57-59]. Subverting NOD2 activity in the vesicle–associated function of NOD2 (i.e. autophagy), SL-1 inevitably activates NOD2-mediated inflammation, which can explain (at least partly) the characteristic inflammation present in mycobacterial infection [60,61].

Mtb lipids are abundantly produced during macrophage infection and are actively trafficked out of mycobacterial phagosomes [62]. Moreover, mycobacterial lipids can be found in extracellular vesicles and could be observed in uninfected ‘bystander’ cells, which expand the bacteria’s sphere of influence beyond the membranes of the infected host cell [62]. For CD pathogenesis, it means that MAP-infected intestinal cells may contaminate with SL-1 surrounding intestinal cells, such as enterochromaffin (EC) cells, Paneth cells, and their progenitor stem cells, all of which are known to be affected in CD [63,64].

In these circumstances, the *NOD2* polymorphism associated with decreased sulfatide recognition makes the host predisposed to mycobacterial infections. When mycobacterial infection is established, the double pressure on the NOD2 vesicle function from the polymorphism and SL-1 substantially increases the chance of defects in acidic vesicle homeostasis. Notably, the 1007 fs NOD2 polymorphism most commonly associated with predisposition to CD, only slightly decreases the NOD2 binding to sulfatide, suggesting that SL-1 presence plays a more important role in CD development than does genetic predisposition [32]. Indeed, only about 5% of NOD2 mutation homozygotes develop CD, suggesting crucial roles for additional factors (like mycobacterial infection) in CD development. Of interest, sulfonated compounds like dextan sulfate and 2,4,6-trinitrobenzenesulfonic acid are used the most frequently for experimental colitis induction.

### Vesicle-associated abnormalities and CD specific features

We found NOD2 in cell-division-specific structures associated with the massive fusion of intracellular vesicles providing the membrane material for cell division [32], [65]. Cell division and a high level of autophagy, where the latter maintains the stemness, are typical stem cell features supported by the expression and functional activities of proteins mediating these processes [66]. NOD2 has an important biological role in bone marrow CD34+ hematopoietic cells [67]. Intestinal crypt Lgr5^+^ stem cells also express Nod2 mediating gut epithelial regeneration [68]. The latter suggests that NOD2 regulates the Notch signaling pathway, a key cell communication pathway that suppresses production of secretory intestinal cells (i.e. EC cells) in favor of higher gut epithelial cell production [69]. Notch activity is promoted by the fusion of Notch receptor-containing endosomes with V-ATPase-containing lysosomes [70-72]. All this suggests that the SL-1-associated unspecific activation of NOD2 in intestinal stem cells may increase production of EC cells, which are responsible for 90% of the body serotonin (5-hydroxytryptamine).

CD-affected intestines have higher numbers of EC cells and levels of serotonin [63,73]. Enteric serotonin is a major gastrointestinal paracrine hormone and neurotransmitter mediating the peristaltic activity, blood clotting and bone metabolism, all impaired in CD [74-78]. The systemic character of serotonin action in the body suggests that imbalances of serotonin in CD can be among the factors mediating the systemic character of the disease. Serotonin imbalances are also found in leprosy and tuberculosis [79,80].

V-ATPase generates the proton membrane potential that is used by vesicular monoamine transporters to sequester newly synthesized or externally up-taken serotonin into intracellular vesicles [81]. SL-1-induced vesicle content leakage will lead to a prolonged exposure of non-sequestered monoamines to cytoplasmic (mitochondrial) monoamine oxidases. This results in an increased conversion of monoamines into toxic aldehydes, causing cell damage and inflammation. These effects in turn enhance EC cell production from intestinal stem cells, making the pathological process self-sustaining [82-84].

### NOD2 and other genetic and non-genetic factors of predisposition to CD

Mechanisms mediating serotonin release from cells become very important when serotonin sequestration is defective. Indeed, genetic polymorphisms of polyspecific organic cation transporters OCTN1/2, translocating cytoplasmic serotonin through the cytoplasmic membrane, are among the CD predisposition factors [85,86]. Moreover, the CD-associated *OCTN1* and *NOD2* gene polymorphisms are additive for CD development [87].

Only about 10 to 20 percent of patients have a family history of CD, suggesting the main role of environmental factors in CD development. Similar to SL-1 competing with sulfatide for NOD2 binding, other factors affecting sulfatide synthesis or accessibility can trigger NOD2 functional deficiency.

CD is more common in urban areas. In general, these areas are better supplied with potable water, which, even after chlorination, may serve as a transmission route of MAP [88]. Chlorate ion (ClO3^−^), often used for or formed as a byproduct in water chlorination, is a well-known inhibitor of PAPS synthesis and consequently sulfatide synthesis. Exposure of cells to sodium chlorate has a similar effect on autophagy as NOD2 deficiency [32]. Thus, the hypothesis of MAP transmission via potable water should include water chlorination as a risk factor.

## Conclusion

The absence of a clear mechanistic explanation of the role of MAP in CD has been one of the main obstacles in transformation of their well-known association into causation. NOD2, an intracellular pattern recognition receptor playing a role in mycobacterial infections and CD, has been suspected as a possible link between them. This review summarizes very recent findings on the NOD2 ligand and functional specificities that establish the causative link between mycobacteria and CD via mycobacteria-specific inhibition of NOD2 function. Moreover, these findings clarify the role of other genetic and environmental factors of predisposition to systemic CD. Further development of these NOD2 findings may provide novel therapeutic targets for CD and other mycobacteria-related pathologies.

## Abbreviations

CD: Crohn’s disease
MAP: *Mycobacterium avium* subsp. *paratuberculosis*
Mtb: *M. tuberculosis*
NOD2: Nucleotide binding and Oligomerization Domain 2
V-ATPase: Vacuolar-type H^+^-ATPase
EC cells: Enterochromaffin cells
PRR: Pattern recognition receptor
GAL3ST1: Galactose-3-O-sulfotransferase 1
DAMP: Damage-associated molecular patterns
LRR: Leucine rich repeats
NACHT: NAIP, CIITA, HET-E, TP-1
CARD: Caspase recruitment domain
OCTN1(2): Organic cation transporter, novel, type 1(2)
PAPS: 3’-phosphoadenosine-5’-phosphosulfate
HIF-1: Hypoxia-inducible factor type 1
